# Selenium biofortification in the 21^st^ century: status and challenges for healthy human nutrition

**DOI:** 10.1007/s11104-020-04635-9

**Published:** 2020-12-03

**Authors:** Michela Schiavon, Serenella Nardi, Francesca dalla Vecchia, Andrea Ertani

**Affiliations:** 1 Dipartimento di Agronomia, Animali, Alimenti, Risorse naturali e Ambiente (DAFNAE), Università di Padova, Viale dell’Università 16, 35020 Legnaro, PD Italy; 2 Dipartimento di Biologia, Università di Padova, Via G. Colombo 3, 35131 Padova, Italy; 3 Dipartimento di Scienze Agrarie, Università di Torino, Via Leonardo da Vinci, 44, 10095 Grugliasco, TO Italy

**Keywords:** Selenium, Nutrition, Health, Biofortification, Phytochemicals, Viral immunity

## Abstract

**Background:**

Selenium (Se) is an essential element for mammals and its deficiency in the diet is a global problem. Plants accumulate Se and thus represent a major source of Se to consumers. Agronomic biofortification intends to enrich crops with Se in order to secure its adequate supply by people.

**Scope:**

The goal of this review is to report the present knowledge of the distribution and processes of Se in soil and at the plant-soil interface, and of Se behaviour inside the plant in terms of biofortification. It aims to unravel the Se metabolic pathways that affect the nutritional value of edible plant products, various Se biofortification strategies in challenging environments, as well as the impact of Se-enriched food on human health.

**Conclusions:**

Agronomic biofortification and breeding are prevalent strategies for battling Se deficiency. Future research addresses nanosized Se biofortification, crop enrichment with multiple micronutrients, microbial-integrated agronomic biofortification, and optimization of Se biofortification in adverse conditions. Biofortified food of superior nutritional quality may be created, enriched with healthy Se-compounds, as well as several other valuable phytochemicals. Whether such a food source might be used as nutritional intervention for recently emerged coronavirus infections is a relevant question that deserves investigation.

## Introduction

Selenium (Se) is a semi-metallic element that possesses chemical and physical characteristics of both non-metals and metals (Boyd, 2011). It is recognized as an indispensable micronutrient for redox biology of many animals and unicellular organisms (Kieliszek 2019). In mammals, Se in the form of the unusual amino acid selenocysteine (SeCys) is incorporated at the catalytic center of selenoproteins via recoding of the opal UGA codon into a SeCys codon (Mangiapane et al. 2014). In humans, at least 25 selenoproteins play important roles in antioxidative systems, hormone balance, immunity, male fertility, resistance to viral infections and cancer prevention (Harthill 2011; Rayman 2012, 2020; Tan et al. 2018; Valea and Georgescu 2018). However, Se exhibits a double-edged behaviour, because it becomes toxic over a threshold concentration established to be 400 µg of Se per day (Institute of medicine 2000; Vinceti et al. 2018). For this reason, similar to boron (B), it has long been termed “*an essential poison*”. Selenium has a relatively narrow safety range as compared to other essential trace nutrients, and thus both the deficiency and toxicity of Se are of worldwide concern (Natasha et al. 2018; Rayman 2020). On the global scale, the Se deficiency and its negative impact on health have progressively increased over recent decades. So far, it has been estimated that 1 billion people have a scanty Se dietary intake (Combs 2001; Tan et al. 2002). This worrisome scenario is likely to worsen in the future because of projected climate changes that will cause reduction of soil Se content, principally in agricultural areas (Jones et al. 2017). Local populations living in low Se areas and feeding on low Se food crops might suffer from Se deficiency disorders because their dietary Se consumption does not meet the daily dose (55–200 µg for adults) recommended to maintain a correct functioning of the metabolism and expression of selenoproteins (USDA–ARS 2012; WHO 2009).

To reduce Se deficiency throughout susceptible regions, the development of high Se crops using biofortification interventions has been accomplished in several countries that are low in Se, such as Finland, United Kingdom, New Zealand, Malawi, parts of China, Tibet, and Brazil (Alfthan et al. 2015; Broadley et al. 2006; Dos Reis et al. 2017; Joy et al. 2019; Wu et al. 2015). Se-biofortified food crops are often enriched in many phytochemicals beneficial to human health, like minerals and antioxidant compounds, which make these foods superior to Se supplementation alone via tablets (D’Amato et al. 2020).

The success of biofortification to enrich plants with Se depends on several variables such as Se species to be used, the mode of Se fertilization, and the crop species. In higher plants, no essential metabolic role of Se has been established and hitherto no specific mechanisms of SeCys incorporation into proteins have been documented (Schiavon and Pilon-Smits 2017a). Selenium is assimilated to Se-amino acids (SeCys and selenomethionine, SeMet) by accessing the metabolic pathway of its analog sulphur (S) (White 2016, 2018). Se-amino acid insertion into proteins in place of S amino acids (cysteine and methionine) can produce malformed proteins causing toxicity (Sabbagh and Van Hoewyk 2012; Van Hoewyk 2013). On the other hand, at low concentration Se can be beneficial to plants. Its positive effects were originally described in Se-hyperaccumulator species (Pilon-Smits et al. 2009). However, further studies with several non-hyperaccumulators have proved that Se promotes the growth, accumulation of beneficial phytochemicals, and antioxidant activity (e.g., Chauhan et al. 2019; Dall’Acqua et al. 2019; Natasha et al. 2018; Schiavon et al. 2013, 2016; D’Amato et al. 2020).

Different approaches can be explored to enrich plants with Se. Some are already widespread or are gaining recognition, while others are limited by *in-situ* restrictions. The aim of this review is to afford a comprehensive overview of the current knowledge of Se biofortification and future research directions. It is focusing on (i) Se distribution in soil and soil properties that may affect the efficacy of Se biofortification strategies; (ii) Se metabolism in plants and possible interferences of Se enrichment with metabolic pathways that might influence the nutritional profile of Se-enriched food; (iii) Se biofortification strategies (conventional and modern agronomic approaches, genetic engineering and plant breeding) and biofortification under challenging conditions; (iv) effects of Se biofortification on plant food nutritional traits; (v) impact of Se-enriched food on human health with special attention to its role in reducing the risk of and treating viral diseases.

## Selenium sources and processes at the plant-soil interface

The geographical distribution of soil Se is very heterogeneous, but Se content in most soils is low and ranges from 0.01 to 2 mg kg^− 1^ on average (world mean 0.4 mg·kg^− 1^) (Fordyce 2013; Oldfield 2002). In tropical soils, soil Se levels are slightly higher and typically range within 2–4.5 mg kg^− 1^ (Mehdi et al. 2013), while seleniferous soils can contain up to 1200 mg Se kg^− 1^ (Fordyce 2013; Oldfield 2002).

Selenium in soils is primarily derived from the parental material of soil. Its content depends on soil type and texture, origin and geological history, mineralogy, organic matter content, weathering degree and rainfalls, dominant soil geogenic processes, and Se deposition (Hartikainen 2005; Mehdi et al. 2013; Wen and Carignan 2007). Natural Se emissions (crustal weathering, sea spray, volcanic plumes) and anthropogenic Se emissions (fossil fuel combustion, non-ferrous metal production and manufacturing) largely contribute to Se deposition, which is affected by vicinity to the coast, altitude and wind directions (Saha et al. 2017; Wen and Carignan 2007).

Low Se soils mainly derive from igneous rocks and are settled in areas characterized by limited atmospheric depositions and elevate erosion rates (Christophersen et al. 2012). Volcanic soils and granite are commonly poor in Se and lay in the mountainous countries of Northern Europe (Mehdi et al. 2013). Conversely, high Se soils originate from sedimentary rocks, generally Cretaceous sediments like black shales, containing selenites and selenides associated with sulphide minerals. Black shales are particularly abundant in Ireland, China (e.g., Enshi in Hubei Province and Ziyang in Shaanxi Province), and arid areas in the western and southwestern states of the USA (e.g. San Joaquin Valley in California), and India (Punjab) (Oldfield 2002; Winkel et al. 2015). Occasionally, high Se contents in soil may depend on the release of toxic levels of geogenically-derived Se in soils and waters caused by human actions (Ohlendorf et al. 2020), on anthropogenic inputs, either industrial or agricultural, or on dust depositions discharged by nearby coal-burning sites (He et al. 2018). Agricultural activities that increase Se levels in soil include irrigation and the long-term use of chemical fertilizers and farmyard manure. Irrigation may elevate Se in soil by either favoring dissolution of Se from minerals rich in Se or bringing Se loads to soil when Se-rich waters are used (Bajaj et al. 2011), while adding Se to chemical fertilizers is a common practice when biofortification is conducted in Se deficient areas, where soil Se is below 0.6 mg kg^− 1^, or when prevailing soil conditions hamper Se availability to plants (Gupta and Gupta 2000).

The specific impact and fate of different Se inputs into agricultural soils have been poorly investigated so far. Further systematic mass balance studies of Se in soils that estimate the amount of Se retained in soil and Se losses through leaching, volatilization and crop removal, are required to better quantify the contribute of each Se source to the content of Se in agroecosystems. These studies might be helpful in view of facing with the undergoing global climate-changes predicted to cause reductions of soil Se in the future (Jones et al. 2017).

**Fig. 1:**
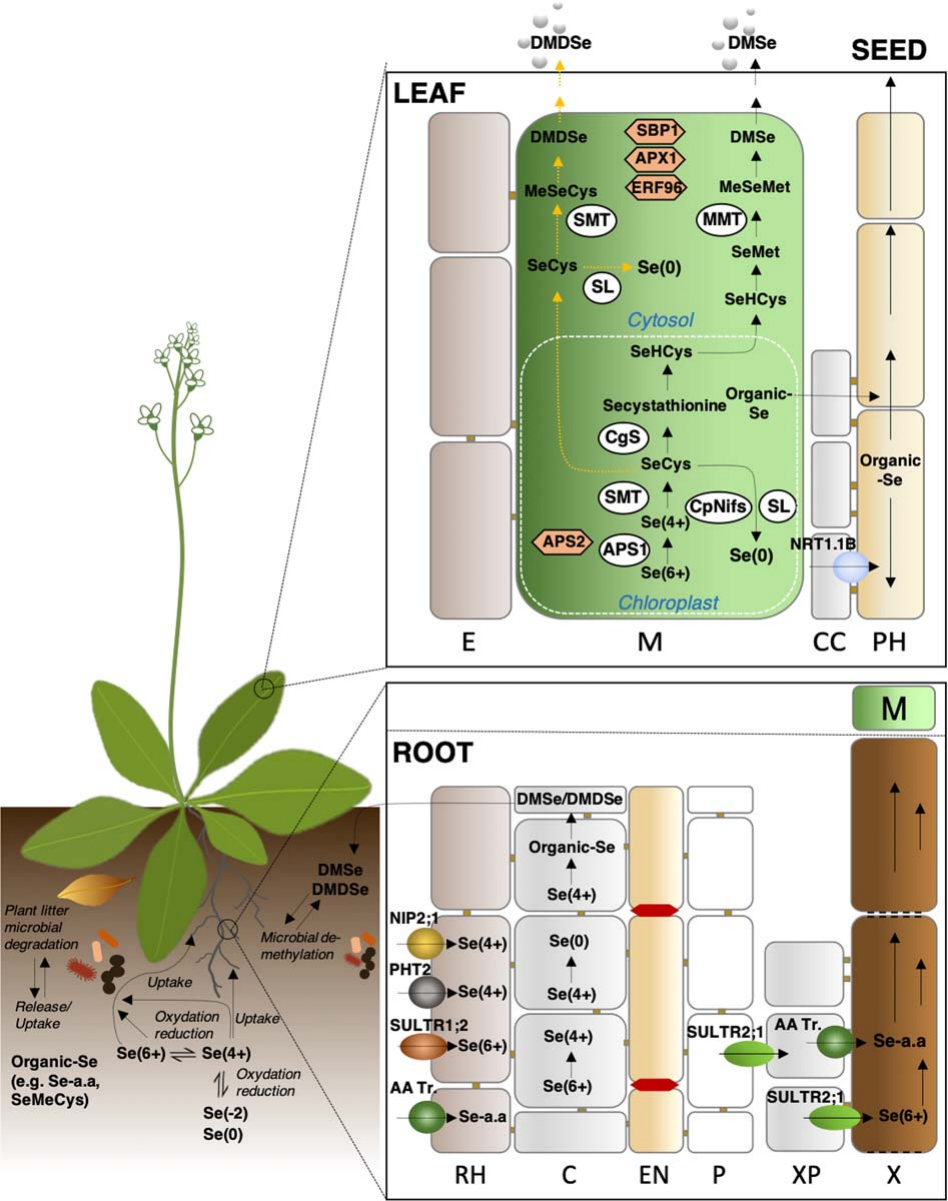
Major pathways of Se at the soil-plant-atmosphere interface, Se movement and partitioning in the plant, with focus on genetic targets for optimization of Se biofortification. Transformation processes in soil are indicated in italics at the corresponding arrows. Soil microorganisms can degrade Se-containing plant litter, thus releasing Se into soil. Se species can be taken up by soil microorganisms or by plants. In the root structure biomagnification, the main transporters involved in Se uptake by plants (SULTR1;2 for selenate, NIP2;1 and PHT2 for selenite, AA Tr. for amino acids) are localized at the rhizodermis (RH). Se metabolism takes place in the root cortical cells (C), and volatile Se species (DMSe and DMDSe) are released into the soil, while Se organic compounds, mainly Se-amino acids (Se a.a.) are transported into the xylem (X) via amino acid permeases (AA Tr.) and delivered to the leaves. DMSe and DMDSe in soil are then de-methylated by rhizosphere microorganisms. Selenate is the main form of Se shuttled through the xylem to the leaves, where it is finally assimilated to Se-amino acids. Selenate is loaded into the xylem by SULTR2;1 localized at the xylem parenchyma cells (XP) and pericycle (P). Both SULTR1;2 and SULTR2;1 from the Se hyperaccumulator *S. pinnata* are currently under investigation. Organic-Se compounds produced in the leaf cells can be loaded into the phloem (PH) and transported throughout the plant. The translocation of SeMet to the seeds is enhanced by overexpression of the NRT1;1B transporter. Volatile DMSe and DMDSe produced in leaves are released into the atmosphere. Enzymes in circles are early targets of genetic engineering for biofortification or phytoremediation, while enzymes in hexagonal shaped boxes are new targets to better explore. Abbreviations: EN: endodermis; E: epidermis; M: mesophyll; CC: companion cells; CgS: Se Cys γ-synthase. For simplicity, *Arabidopsis thaliana* has been used as a model species in figure

Selenium in soils is found under inorganic and organic forms, where it holds different oxidation states ranging from − 2 to + 6. Se solubility and mobility in soil increase with increasing oxidizing conditions (high redox potential). In this regard, selenate (SeO_4_^2−^) is the most water-soluble, mobile and bioavailable inorganic Se species in oxic soils, with low adsorption affinity to oxide surfaces (Hartikainen 2005). Under low soil redox potential conditions, selenate can be reduced to selenite (SeO_3_^2−^), whose compounds are typically the most abundant Se species in anoxic soils (Fig. 1) (Shahid et al. 2018). Selenite is less mobile and bioavailable than selenate owing to its tendency to be adsorbed on oxide surfaces at low pH (Hartikainen 2005). Therefore, available soil Se is usually poorly correlated with total soil Se. Under intense reducing conditions, selenite can be further reduced to elemental Se (Se^0^) or selenides (Se^2−^) (Hartikainen 2005, Winkel et al. 2015). Bacteria are reported to produce (nano)-sized Se(0) from selenate and selenite (Juárez-Maldonado et al. 2019; Ni et al. 2015), while Se in the − 2 oxidation state can exist as hydrogen selenide or metallic selenides, which are very insoluble forms of Se (Winkel et al. 2015).

Generally, soil organic forms of Se occur as complexes with organic matter and combined with organic or organo-mineral colloids (Hartikainen 2005). Organo-Se compounds include methylated or unmethylated Se-amino acids, and volatile Se forms (dimethyl selenide, DMSe, and dimethyl diselenide, DMDSe), but a large fraction of them is still undisclosed (Winkel et al. 2015) (Fig. 1). These compounds can either derive from decomposition processes of plant and microbial biomass or from addition of Se-enriched plant material (green manure) to soil when biofortification of crops with Se is attained.

Several factors control Se mobility and solubility in soils such as pH, sorption, and desorption reactions, redox potential, organic/inorganic compounds, and dissolution processes in soils and sediments. Sorption-interactions depend on the amount of sorption components and are largely governed by electrostatic forces, which in turn rely on soil pH. When Se in soil prevails in anionic form, it can be retain by sorption on oxides and hydroxides (e.g. iron or aluminum oxides/hydroxides), or anionic clays by electrostatic interactions (Eich-Greatorex et al. 2007; Winkel et al. 2015). Se adsorption rate increases while decreasing the pH, as electrostatic interactions become sturdier (Eich-Greatorex et al. 2007). On the other hand, raising in pH or reducing clay and iron (or aluminum) oxide/hydroxide content in the soil result in higher Se mobility and bioavailability.

Soil organic matter (OM) is another critical factor altering soil Se availability, as soils rich in OM commonly display higher Se retention capacity (Fordyce, 2013; Winkel et al. 2015). OM can reduce Se available in soil by direct complexing with Se. Otherwise, indirect complexation of Se with OM-metal complexes or microbial reduction and incorporation into organic compounds (e.g. amino acids, proteins) may occur in soil (Winkel at al. 2015). From either organic complexes or compounds, Se can be promptly mobilized or retained depending on the type of binding.

## Selenium uptake and assimilation pathways in plants

Plants accumulate Se in their organs depending on Se phytoavailable in soil, with a large variation of shoot Se concentration between genera, species and even ecotypes within species (Mehdi et al. 2013; Schiavon and Pilon-Smits 2017a; White 2016, 2018).

Plants are distinguished in major ecological groups depending on their capacity to accumulate Se in the shoot when growing in their natural environment (White 2016). Se-hyperaccumulator species thriving on seleniferous soils generally contain and tolerate extremely high Se concentrations (over 1000 µg Se g^− 1^ d.wt.) that could eventually be toxic to grazers, whereas non-hyperaccumulator species are Se-sensitive and thereby accumulate less than 100 µg Se g^− 1^ d.wt. (non-accumulators) or up to 1000 µg Se g^− 1^ d.wt. (accumulators or indicators) (Schiavon and Pilon-Smits 2017a, b; White 2016). Hyperaccumulators store Se mainly in the form of methylselenocysteine (MeSeCys) and selenocystathionine, while SeMet is the main Se organic compound identified in non-hyperaccumulators (Pilon-Smits, 2019; Schiavon and Pilon-Smits 2017a, b).

Plants take up both inorganic (selenate, selenite and elemental Se) and organic (e.g. Se-amino acids) Se species, but not selenides (Chauhan et al. 2019; Gupta and Gupta 2017; Schiavon and Pilon-Smits 2017a; White 2016) or colloidal elemental Se (White 2016, 2018). Their capacity to take up Se is apparently higher for organic over inorganic species (Kikkert et al. 2013). Se-amino acids in particular, are likely to enter the plant cells via broad specificity amino acid transporters (Fig. 1) (Lima et al. 2018). Though, selenate is the main form of Se taken up by plants, and its transport across cell membranes is an energy-dependent process mediated by the sulphate transport system (Lima et al. 2018; Schiavon and Pilon-Smits 2017a; White 2018). Competition events in soil between sulphate and selenate, as well as plant sulphate transporters (SULTR) differing in affinity for these two anions, influence the rate of selenate uptake by plants (El Mehdawi et al. 2018; White et al. 2004, White 2016). Selenite compounds instead, are conveyed over plant cell membranes via phosphorus (P) and silicon (Si) transporters, with differences between selenite anion (SeO_3_^2−^), hydrogenselenite ion (HSeO_3_^−^), and selenous acid (H_2_SeO_3_) (Wang et al. 2019; Zhang et al. 2014). Precisely, H_2_SeO_3_ is transported by Si transporters (LSI1) and aquaporins (OsNIP2;1) (Wang et al. 2019; Zhao (FJ) et al. 2010, b), while HSeO_3_^−^ and part of SeO_3_^2−^ mainly use low- and high-affinity P transporters (OsPT2), respectively (Zhang et al. 2014).

**Fig. 2:**
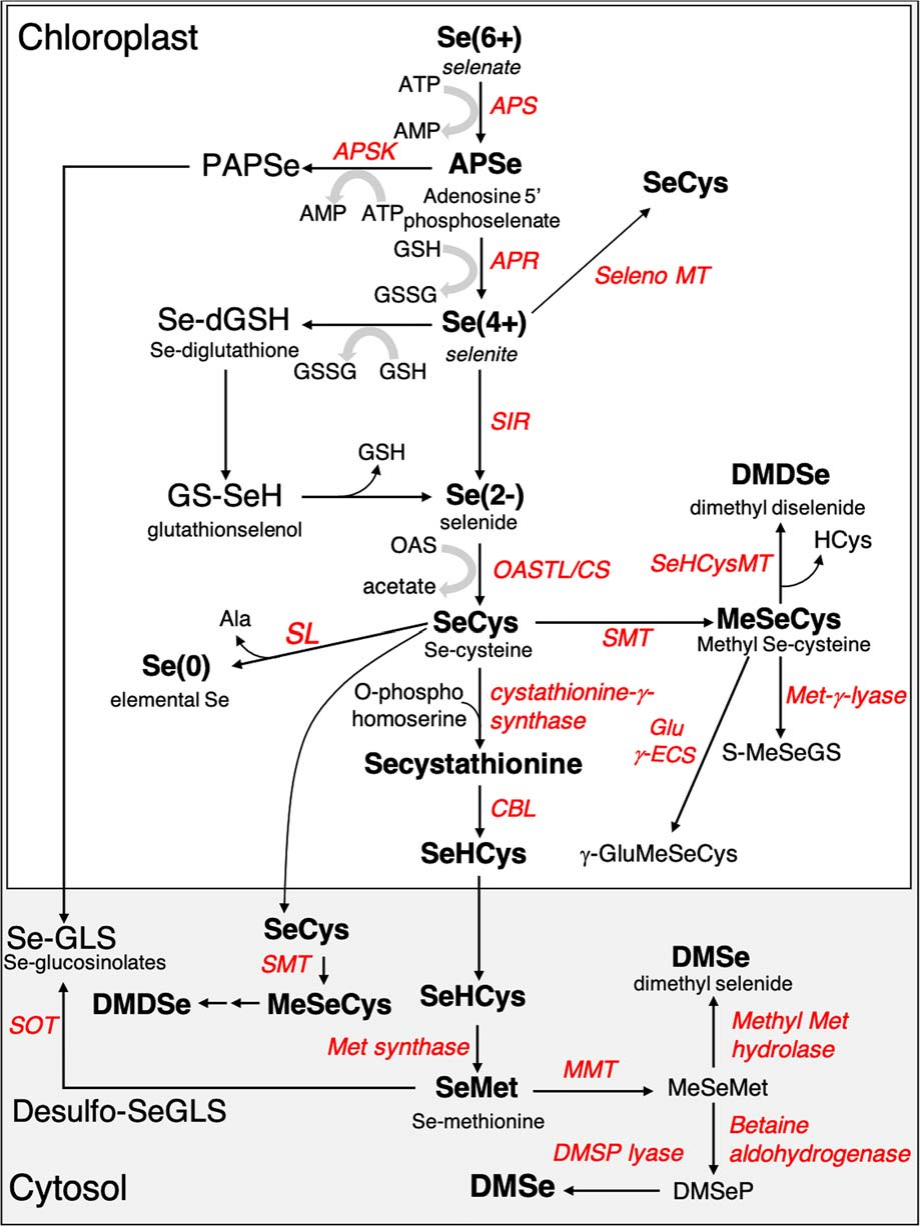
Schematic diagram of the biochemical reactions of Se assimilation and their subcellular compartmentalization in plants. Enzymes are indicated in italics, major Se compounds and intermediates are indicated in bold. Information was compiled from previous reviews (Chauhan et al. 2019; Schiavon and Pilon-Smits 2017a; Sors et al. 2005; White 2016, 2018). Enzymes: APS: ATP-sulphurylase; APSK: APR: APS reductase; APS kinase; SIR: selenite reductase; CBL: cystathionine β-lyase; SMT: Se cysteine methyltransferase; SeHCysMT: Se-Homocysteine methyltransferase; MT-γ-lyase: methyl Se cysteine-γ-lyase; SL: Se cysteine lyase; MMT: Methionine methyltransferase; OASTL/CS: O-acetylserine thiol-lyase/Cysteine synthase; SOT: sulfotransferase; DMSP lyase: dimethylselenopropionate lyase; Glu-γ-ECS: γ-glutamyl cysteine synthetase. Compounds: GSH/GSSS: reduced/oxidized glutathione; PAPSe: phosphoadenosine 5’- phosphoselenate; OAS: O-acetylserine; Ala: alanine; MeSeMet: Methyl Se methionine; DMSP: dimethylselenopropionate; γ-GluMeSeCys: γ-glutamylmethylselenocysteine; S-MeSeGS: S-Methyl-selenoglutathione; HCys: homocysteine

Once entered the root cells, inorganic Se is delivered to the plastids where proceeds along the S assimilation pathway to produce SeCys and SeMet (Fig. 2) (Chauhan et al. 2019; Schiavon and Pilon-Smits 2017a; Sors et al. 2005; White 2016, 2018). If selenate is taken up by plants, it must be activated before reduction to selenite. Selenate activation is a rate-limiting step mediated by the ATP sulphurylase enzyme (APS), which couples selenate to ATP to generate adenosine 5′- phosphoselenate (APSe) (Pilon-Smits et al. 2009). APSe reduction to selenite is then conducted by the APS reductase enzyme (APR), via transfer of two electrons from glutathione (GSH). In a parallel pathway, APSe can be phosphorylated to produce 3′-phosphoadenosine 5′-phosphoselenate (PAPSe), which is used as a substrate for sulfation of desulfo-glucosinolates (Fig. 2). Selenite can be reduced enzymatically by the activity of the sulfite reductase enzyme (SiR) to produce selenide, which is incorporated into SeCys by the enzyme complex cysteine synthase, containing both serine acetyl transferase (SAT) and O-acetylserine (thiol) lyase (OAS-TL) enzymes (White 2018). Alternatively, selenite reduction can be realized through a non-enzymatic two step-reaction, where selenite is initially converted to selenodiglutathione (GS-Se-SG) in the presence of GSH. GS-Se-SG is further reduced to selenopersulfide/glutathionylselenol (GS-SeH), which reacts with O-acetylserine (OAS) to generate SeCys (White 2016, 2018). Otherwise, SeCys can be formed directly from selenite by the activity of the selenomethyltransferase enzyme (SMT) (Chauhan et al. 2019) The synthesis of SeMet from SeCys takes place partly in the cytosol and requires selenocystathionine and selenohomocysteine to be formed as intermediates (Fig. 2). This biochemical transformation involves three enzymes working in series: cystathionine γ-synthase (CGS), which promotes the formation of Se-cystathionine by condensing O-phosphohomoserine (OPH) and SeCys; cystathionine β-lyase (CBL) and methionine synthase (MS) (Sors et al. 2005; White 2018).

Incidentally, Se-amino acids can be integrated into proteins, thus disrupting their molecular folding and impairing their function (Sabbagh and Van Hoewyk 2012; Van Hoewyk 2013). The magnitude of this event seems to be more related to the Se/S ratio in plant tissues than the Se content alone (White et al. 2004). To prevent toxicity derived from Se amino acid misincorporation in proteins, SeCys and SeMet can be methylated to produce methyl-SeCys (MeSeCys) or methyl-SeMet (MeSeMet), respectively, which are further converted into volatile DMSe (in non-hyperaccumulators) and DMDSe (in hyperaccumulators) (Pilon-Smits and LeDuc 2009; Van Hoewyk 2013; White 2016). SeCys can also be converted to elemental Se (Se^0^) and alanine via a reaction ruled by the enzyme SeCys-lyase (SL) (Pilon-Smits and LeDuc 2009), while MeSeCys could be conjugated with glutamate by the enzyme γ-glutamylcysteinesynthetase to form γ-glutamylmethyl-SeCys (γ-GluMeSeCys) (Fig. 2) (White 2018).

## How can crops be enriched with selenium? Conventional and novel approaches

A deep knowledge of Se biogeochemistry, uptake mechanisms and assimilation by plants is a mandatory prerequisite for Se biofortification of food crops. Biofortification is the process of adding essential micronutrients and other health-promoting compounds to food crops with the aim to improve the nutritional quality of diets ultimately consumed by people (Garg et al. 2018; Jha and Warkentin 2020; Schiavon and Pilon-Smits 2017b; White and Broadley 2009; Wu et al. 2015; Zhao and McGrath 2009). It is an innovative and reasonably easy practice to manage, affordable and effective in the long-term to tackling micronutrient malnutrition (Ros et al. 2016; White and Broadley 2009). Successful outcomes depend on environmental and economical characteristics of local food systems, but also on their acceptance by farmers and populations that feed on biofortified food. To get acceptance, biofortified crops must be staple, high yielding and worthwhile to be adopted by farmers, and most consumers in target populations must consume the biofortified food in amounts enough to measurably improve their nutritional status (Miller and Welch 2013).

**Fig. 3:**
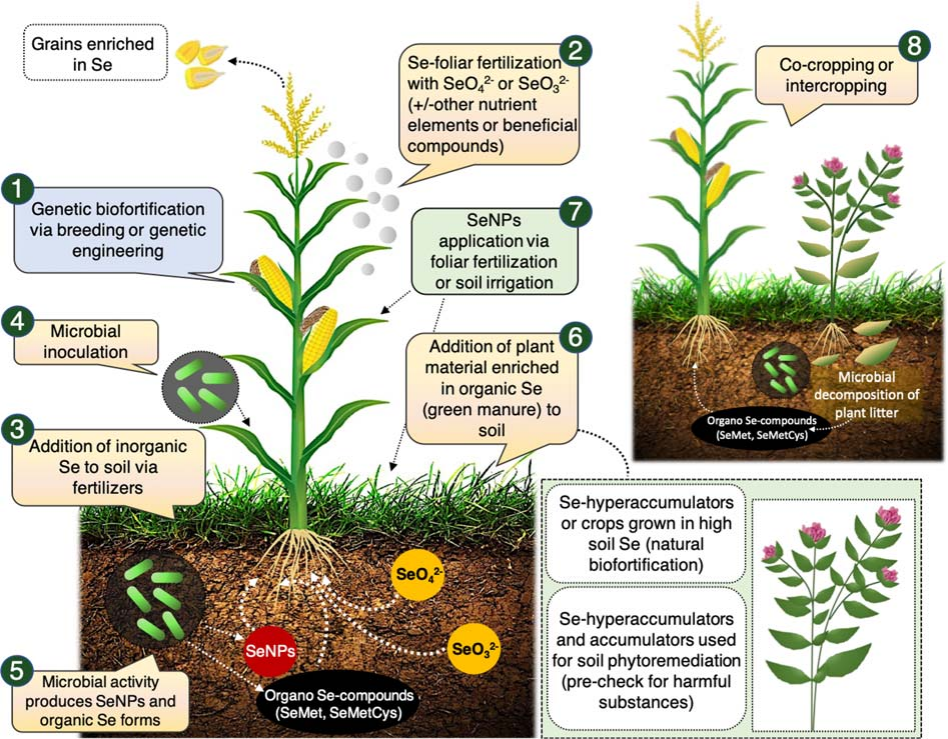
Overview of biofortification strategies that can be exploited to enrich crops in selenium. Se biofortification can be achieved using genetic tools (1), via conventional or assisted breeding and genetic engineering, or through fertilization with Se-fertilizers via foliar application (2) or soil amendment (3). Agronomic biofortification can be integrated by the use of rhizosphere or endophytic microorganisms, either inoculated into plants (4) or applied to soil (5). Alternatively, plant material enriched with Se-organic compounds from hyperaccumulators or accumulators used in phytoremediation, or from crops grown in naturally enriched soils can be used as green manure (6). Nanosized biofortification is performed using SeNPs applied to leaves or soil (7). Co-cropping or intercropping practices use Se-hyperaccumulators to enrich soil with Se-organic compounds promptly available for uptake by neighboring plants or succeeding crops (8)

Several studies have been conducted to generate Se-enriched functional food and various biofortification interventions have been developed and tentatively optimized using agronomic approaches, genetic engineering and plant breeding (Bañuelos et al. 2017; Cakmak, 2008; D’Amato et al. 2020; Ros et al. 2016; Schiavon and Pilon-Smits 2017b; White 2016; Zhu et al. 2009). The addition of Se compounds to food during its processing (process fortification) carried out by the food industry has also been proposed as an effective practice in alternative to agronomic biofortification performed using Se fertilizers (Haug et al., 2007). Biofortification strategies, summarized in Fig. 3, are influenced by several variables such as method of Se supplementation, Se dose and species, soil Se, cropping systems and tillage practices, environmental and weathering conditions, crop species/variety, plant growth stage and joint application with other micronutrients (Bañuelos et al. 2017; D’Amato et al. 2020; Dall’Acqua et al. 2019; Hawrylak-Nowak, 2013; Schiavon et al. 2013, 2016). So far, most research dealing with Se biofortification has targeted at the application of Se alone or occasionally combined with only one micronutrient, generally silicon, iodine (I) or zinc (Zn) (Golubkina et al. 2018; Sattar et al. 2017; Cakmak et al. 2020; Golob et al. 2020). Only few studies have addressed crop biofortification with multiple micronutrients (Mao et al. 2014; Zou et al. 2019), although deficiency of multiple trace nutrients is an issue of global concern. A recent field experiment managed in six different countries (China, India, Mexico, Pakistan, South Africa, and Turkey) varying in soil type and environmental conditions, proved that foliar fertilization of wheat (*Triticum aestivum* L.) plants with a blend of Se, Fe, I, and Zn, was effective in enriching grains with all four elements (Zou et al. 2019). Based on these promising results, future Se biofortification research should address the feasibility of supplementing crops with cocktails of micronutrients without unwanted crop yield trade-off.

### Agronomic biofortification

Agronomic biofortification is commonly employed in low Se areas and mainly consists in Se addition to soil and Se foliar fertilization, typically realized using selenate- or selenite-based fertilizers (Alfthan et al. 2015; Broadley et al. 2006; Wu et al. 2015). Selenium fertilizers are generally employed in small amounts (10–20 g Se ha^− 1^) to achieve biofortification goals. Therefore, to ease their application, they are frequently mixed into other commercial nutrient fertilizers (e.g., mix of nutrients, urea, calcium nitrate) functioning as “carriers” of Se (Premarathna et al. 2012; Ramkissoon et al. 2019). The addition of organic acids to Se fertilizers can further promote Se chelation with organic compounds, thus causing incremental plant uptake of Se and efficiency of Se fertilizers.

The application of Se fertilizers to soil commonly results in increased total and bioavailable Se (Broadley et al. 2010), and thus in higher Se concentration in crop edible products (Chilimba et al. 2012; Curtin et al. 2006; Lyons, 2010; Poblaciones et al. 2014). Such a practice is apparently not associated with environmental risks of Se leaching into groundwater, being this process limited by Se binding to soil organic matter and positively charged sites, and Se losses via volatilization (De Feudis et al. 2019). Field tests have been successfully conducted by applying Se to soil in Finland (Alfthan et al. 2015; Hartikainen, 2005), UK (Broadley et al. 2010; Lyons, 2010), and New Zealand (Curtin et al. 2006). Nevertheless, the efficacy of this approach can be limited by heterogenous distribution of Se in soil and by those soil conditions that control Se speciation and uptake by plants such as pH, organic matter, oxygenation, content of competing ions, soil age, chemical and biological Se transformations (Bañuelos et al. 2017; Duncan et al. 2017; Haug et al., 2007; Schiavon and Pilon-Smits, 2017b; Stroud et al. 2010). It has been estimated that only 12% of soil-applied Se fertilizers is taken up by plants on average, as most of Se is fixed and retained in the soil, and thus is not bioavailable (Broadley et al. 2010). Negligible residual Se is then available for succeeding crops, and thus Se fertilizers must be applied to soil for each growth period. In contrast, foliar Se fertilization is up to 8 times more effective than soil Se supplementation (Ros et al. 2016). This is likely because of more rapid Se uptake and assimilation processes, no need of Se root-to-shoot translocation to the edible parts of crops, and prevention of Se losses due to immobilization of Se compounds in soil (Ramkissoon et al. 2019).

However, soil amendment with Se can determine positive shifts in the amount and diversity of soil microbial communities, by increasing the relative abundance of plant growth promoting rhizobacteria (PGPR) and reducing the occurrence of pathogenic fungi (Liu et al. 2019). Beneficial rhizosphere microorganisms could enhance soil Se phytoavailability and the plant Se use efficiency of applied fertilizers (Durán et al. 2014; Lindblom et al. 2014; Mora et al. 2015; Yasin et al. 2015a,b) owing to their capacity to reduce oxidized and methylated forms of Se (Winkel et al. 2015) and increase the volume of soil explored by the plant roots to acquire Se (White and Broadley 2009). The addition of beneficial microorganisms to soil or the inoculation of plants with PGPR might enhance Se biofortification of crops. For instance, the inoculation of wheat plants with specific microorganisms, alone or combined with arbuscular mycorrhizal fungi (AFM), was effective in increasing Se concentration in their edible products (Duran et al. 2013; Yasin et al. 2015a,b). Similarly, inoculating shallot bulbs with AFM increased selenocystine and selenate contents by 36% and 21%, respectively, compared to non-inoculated plants (Golubkina et al. 2019).

Crop enrichment with Se could also be achieved by combining different types of Se phytotechnologies (Bañuelos et al. 2015; Schiavon and Pilon-Smits 2017b; Stonehouse et al. 2020). Se-laden plant material derived from plants employed in Se phytoremediation could be recycled as green fertilizer to increase Se content in agricultural soils or as a supplemental fodder for livestock (Bañuelos et al. 2009). In this case, before being introduced into the food web, the plant material must be carefully checked for its content in hazardous elements, as soils intended to be remediated are often contaminated with many metal/loids. Alternatively, plant material derived from Se-hyperaccumulators thriving on seleniferous soils and containing high levels of Se, especially in organic forms (e.g. SeMet, MetSeCys), could be used as green manure (Bañuelos et al. 2015, 2017; Wan et al. 2018). Crops to be amended with this material may be selected via breeding or engineered for preferential uptake and accumulation of organic Se in edible products. Such an approach might have relevant implications for healthy nutrition, because organic Se compounds in food may be more quickly used than inorganic Se by enzymes that support antioxidant activities in humans and animals (Davis 2012). Se-hyperaccumulators could be otherwise used in co-cropping or intercropping practices. In this case, they will deposit their leaf litter on soil at the end of their growing season, and thus the soil will be enriched with Se organic compounds available for the uptake by the neighboring crops.

Growing crops is soils naturally rich in Se or irrigated with waters naturally abundant in Se could be another Se biofortification option to explore. This practice is known as “*natural biofortification*” and is gaining interest in certain areas worldwide (Wu et al. 2015). It has been realized in some regions of China (Dinh et al. 2018), USA (Bañuelos et al. 2015, 2019) and India (Dhillon and Dhillon 2009), but the number of studies conducted in the field environment is still limited so far, as most research has been performed under controlled and simplified environments, with homogeneous soil properties that do not resemble the real soil conditions of agroecosystems. One drawback of this practice is that agricultural use of seleniferous soils can hasten the release of Se in the environment under certain conditions, particularly when the Se gets concentrated by evapotranspiration, thus generating possible eco-toxicological hazard.

### Biofortification by using nano-sized selenium

In very recent years, emerging cutting-edge technologies have advised the application of Se in the form of Se nanoparticles (SeNPs) in alternative to conventional Se fertilizers for enriching crops with Se-organic compounds (Babajani et al. 2019; Juárez-Maldonado et al., 2019). SeNPs vary in shape and size and can be synthesized from Se salts precursors, mainly selenite and selenate, in the presence of reducing agents (e.g. proteins, phenols, alcohols and amines) produced by bacteria, fungi and plant extracts (Husen and Siddiqi 2014; Nayantara and Kaur 2018). The biogenic conversion of Se salts to zerovalent SeNPs by Se specialist bacteria has been described in a number of studies (Hunter and Manter 2008; Hunter 2014; Ni et al. 2015), but a great task of scientists remains the synthesis of clonable SeNPs. Indeed, the effects of nanoparticles (NPs) on plants, beyond depending on NP concentration and mode of application (foliar, substrate, seeds), are strongly affected by the method handled for their synthesis, which is further responsible for NP specific properties (size, shape). Recently, *Pseudomonas moraviensis stanleyae*, a bacterium isolated from the roots of the Se hyperaccumulator *Stanleya pinnata*, showed to tolerate extremely high Se concentrations and produce intracellular SeNPs, and its glutathione reductase enzyme has been proposed as a good candidate for the synthesis of clonable SeNPs (Ni et al. 2015).

Harnessing SeNPs as Se-nanofertilizers holds the potential for synchronized Se management in terms of release and uptake by crops, while preventing Se losses in agroecosystems that may occur when commercial Se fertilizers containing selenate and selenite salts are employed (Babajani et al. 2019). SeNPs seem to be less toxic to plants than selenate and selenite salts, as reported in tobacco (*Nicotiana tabacum* L.) (Domokos-Szabolcsy et al. 2012), garlic (*Allium sativum* L.) (Li et al. 2020) and *Vigna radiata* plants (Bărbieru et al. 2019). They can also improve the quality traits of vegetables, as described in strawberry (*Fragaria* × *ananassa*) and tomato (*Solanum Lycopersicon* L.), whose fruits were more enriched in organic acids (e.g., malic, citric and succinic acids) and sugars (e.g. glucose, fructose and sucrose) after treatment with SeNPs (Morales-Espinoza et al. 2019; Zahedi et al. 2019). However, SeNPs may induce toxicity at high levels (Hussein et al. 2019). For instance, in groundnut (*Arachis hypogaea* L.) plants, low doses of SeNPs improved yield components and seed oil, but high doses altered the protein profile and fatty acid composition by increasing unsaturated fatty acids and/or decreasing saturated fatty acids compared to untreated plants (Hussein et al. 2019).

Despite the advantages offered by Se-agronanotechnologies, insufficient literature exists on SeNPs action in plants and their interactions with plant ecological partners, transformation in the food web and the environment. SeNPs are largely used in pharmaceutical applications to increase the bioavailability of drugs and targeting therapeutic agents to specific organs, and are envisioned as next-generation safe ingredients for dietary supplements because they can gradually release Se and better control targeted mechanisms of actions, with only few side effects (Constantinescu-Aruxandei et al. 2018). Though, the release of SeNPs and their fate in the environment following biofortification may pose concerns, cause the lack of information about the impact of SeNPs on public safety and their potential toxicity. It must be noted that SeNPs are becoming emerging contaminants in some areas worldwide due to their intense use in electronics and material productions (Rashid et al. 2016). Therefore, to avoid any unpredictable health hazard from SeNPs occurrence in the environment, further studies should systematically address SeNPs risk assessment and management when used to agricultural purposes.

### Biofortification using plant genetics

Selenium biofortification through conventional breeding is likely to be the most established, sustainable and long-term method of crop biofortification (White and Broadley 2009). It is termed “*genetic biofortification*” and aims at selecting plant cultivars of high/moderate capacity to take up and translocate Se to the edible parts, or having preferential uptake of organic Se (SeMet and/or MetSeCys) (Bañuelos et al. 2017; Broadley et al. 2006; Wu et al. 2015; Zhu et al. 2009). Breeding of crops with high ability to accumulate Se is a feasible practice because enough inter- and intraspecies genetic variation exists in grain Se concentration of several cereal and leguminous crops, as well as in onion bulbs (*Allium cepa* L.), Brassicaceae *spp*., chicory (*Cichorium intybus* L.), lettuce (*Lactuca sativa* L), tomato (*Solanum lycopersicum* L.) and pepper (*Capsicum annuum* L.) fruits, and potato (*Solanum tuberosum* L.) tubers (for specific references see review from White 2016). In this regard, it has been speculated that soil Se phytoavailability and environmental factors are primary factors in controlling Se concentration in edible parts, especially in cereal grains, and that genotypic variation of Se concentration might become more relevant under high Se phytoavailability (Zhu et al. 2009).

So far, a number of chromosomal loci (QTLs) associated with high Se accumulation in grains and leaves of several crops have been identified (Ates et al. 2016; Norton et al. 2010; Wang et al. 2017; White 2016; Zhang et al. 2006). Selecting plant cultivars of high Se concentration in the edible products can be used in marker-assisted breeding (MAB) to transfer these high-Se QTLs to high-yielding low Se cultivars (Pilon-Smits and LeDuc 2009; Wu et al. 2015). One main limitation in the plant breeding, either conventional or marked assisted, is that it must be integrated by agronomic biofortification interventions using Se fertilizers when crops are grown in low Se areas.

The advent of modern molecular tools and analytical technologies has brought to advances in research focusing on Se biofortification for designing future and more effective strategies. Analytical methods include synchrotron X-ray fluorescence and X-ray absorption near edge structure spectroscopies principally, while molecular technologies benefit from high-speed and low-cost of next generation sequencing (NGS), and encompass oligo-directed mutagenesis, reverse breeding, RNA-directed DNA methylation, and DNA editing (Carvalho and Vasconcelos 2013). Such technologies, along with functional genomics gene technology, might be supplementary tools to breeding and genetic engineering (Hung et al. 2012; Pilon-Smits and LeDuc 2009; Wang et al. 2018). However, genetic engineering is far from being widespread and accepted compared to agronomic biofortification and conventional breeding because of imperative restrictions in the use of transgenics yet existing in many countries (Zhu et al. 2009).

A few transgenics suitable for biofortification have been generated so far, with increased capacity to acquire and accumulate Se, preferentially in organic form, in the edible products (Pilon-Smits and LeDuc 2009; White and Broadley 2009; Zhu et al. 2009). Most of these transgenics overexpress sulphate transporters, or enzymes that catalyze rate-limiting steps in Se assimilation such as ATP-sulphurylase, or are involved in mechanisms that prevent Se misincorporation into proteins, like selenocysteine lyase and selenocysteine methyltransferase (Zhu et al. 2009).

Other genes have been successfully targeted by genetic engineering in the last decade, with positive outcomes for Se biofortification. For instance, the overexpression of the selenium binding protein gene SBP1 in *Arabidopsis thaliana* enhanced the resistance of plants to selenite via a GSH-dependent mechanism (Agalou et al. 2005). Similarly, the loss-of‐function mutations in the gene APX1 coding for a cytosolic ascorbate peroxidase enzyme or the overexpression of the Ethylene response factor ERF96 improved Se tolerance and accumulation in *A. thaliana* (Jiang et al. 2016,2020). In the Se accumulator *Brassica juncea* L., a novel selenocysteine methyltransferase enzyme has been identified, which is capable to methylate both homocysteine and SeCys substrates (Chen et al. 2019). The overexpression of this enzyme in tobacco plants increased total Se and MeSeCys accumulation (Chen et al. 2019). Another potential gene target is the NRT1.1B transporter, a member of the rice peptide transporter (PTR) family involved in nitrate transport, because it displays SeMet transport activity and its overexpression in rice leads to higher SeMet accumulation in grains (Zhang et al. 2019).

New targets for genetic manipulation have also been identified in the Se-hyperaccumulator *Stanleya pinnata*, such as selenate/sulphate transporters (El Mehdawi et al. 2018) and one isoform of ATP-sulphurylase (APS2) (Jiang et al. 2018). *S. pinnata* owns a root high affinity sulphate/selenate transporter, SULTR1;2, which is constitutively highly expressed and does not undergo the canonical repression operated by high sulphate in non-hyperaccumulators (El Mehdawi et al. 2018; Wang et al. 2018). In addition, this hyperaccumulator shows elevate expression of the low affinity sulphate transporter SULTR2;1, which plays a role in sulphate/selenate root to shoot shuttling. Both SULTR transporters could be potential candidates of transgenic technologies for the generation of high Se crops. The APS2 isoform of *S. pinnata* instead, is a quite unique and fascinating enzyme, as its expression exclusively localizes to the cytosol (Jiang et al. 2018). Normally, APS isoforms work in the plastids for S/Se assimilation and the APS2 isoform from two Se non-hyperaccumulators, *A. thaliana* and *Stanleya elata*, has a dual localization, plastidial and cytosolic (Bohrer et al. 2015; Jiang et al. 2018). The elevate expression of APS2 in *S. pinnata* might be responsible for its Se hypertolerance. For this reason, APS2 function is currently under investigation.

Early and novel targets of genetic engineering are indicated in Fig. 1.

## Selenium biofortification in the context of global environmental challenges

Plants often face with adverse environmental constraints, which are mostly the result of anthropogenic activities and global climate changes (Mall et al. 2017). Soil salinity, drought and heat stress in particular, have a negative impact on sustainable agricultural systems and severely impair crop yields, especially in arid and semi-arid regions where rainfalls are scarce. Plants growing under such conditions suffer from osmotic stress along with oxidative stress due to increased production of reactive oxygen species (ROS) (Fahad et al. 2017). In this regard, stressed crops might get benefits from Se biofortification, Se acting as a direct or indirect antioxidant (Natasha et al. 2018).

Overall, the beneficial effects triggered by Se are primarily associated to its dose and the capacity of plants to accumulate and tolerate Se (Hawrylak-Nowak 2013; Ríos et al. 2010; Schiavon et al. 2013). At low doses Se promotes the reduction in electrolytic leakage and the recovery of cell integrity under various stress conditions (Zembala et al. 2010; Malik et al. 2012), and enhances the cell antioxidative capacity by stimulating the activity of antioxidant enzymes (superoxide dismutase, SOD, catalase, CAT, peroxidase, POD, ascorbate peroxidase, APX, monodehydroascorbate reductase, MDHAR, dehydroascorbate reductase, DHAR, glutathione peroxidase, GPX, glutathione reductase, GR, and glutathione-S-transferase, GST) and the synthesis of antioxidant metabolites (e.g., ascorbic acid, AsA, glutathione, GSH), as a mechanism for mitigating the effects of ROS (Hasanuzzaman and Fujita 2011; Hasanuzzaman et al. 2011; Jiang et al. 2017; Pilon-Smits et al. 2009). Indeed, at low levels ROS mediate the signal transmission in the defense responses of cells under stress, but at high levels they injure cell components. Therefore, the regulation of the antioxidant system by Se may lead to the reduction of ROS and, in this way, changes their role from detrimental to beneficial. In addition, low Se has been reported to delay plant senescence (Hajiboland et al. 2019), which can be induced prematurely by stress conditions causing reduction of crop yields. In this respect, Se modulates the levels of nitric oxide (NO) in plants (Hajiboland et al. 2019), which in turn negatively regulates several elements (receptors, signal transduction proteins and/or transcription factors) involved in ethylene production and senescence processes (Freschi 2013).

Nevertheless, at high doses Se provokes the synthesis of malformed proteins, reacts with thiol groups and GSH to induce ROS over-generation (Gosh and Biswas 2017; Natasha et al. 2018; Van Hoewyk 2013), and disrupts reactive nitrogen species (RNS) resulting in protein tyrosine nitration (Gupta and Gupta 2017; Kolbert et al. 2018). During high Se condition, GSH can be possibly depleted because of Se/S competition for uptake and assimilation, thereby enhancing ROS accumulation (Dall’Acqua et al. 2019; Van Hoewyk 2013).

Typical beneficial effects of Se described in food crops growing in salinized environments generally include the improvement of agronomic traits (e.g. shoot length, shoot diameter, fresh and dry biomass) (Diao et al. 2014; Hawrylak-Nowak 2009; Hasanuzzaman et al. 2011; Jiang et al. 2017), the increase of net photosynthesis and water contents (Diao et al. 2014; Habibi 2017; Hawrylak-Nowak 2009; Jiang et al. 2017), the stimulation of antioxidant processes and accumulation of non-enzymatic antioxidants (AsA GSH, phenolic compounds), accumulation of osmolytes (proline, sugars and K^+^), the reduction of lipid peroxidation and ROS concentration in cells, and the regulation of Na^+^ homeostasis through increased expression of genes encoding Na^+^/H^+^ antiporters (NHX), as reported in maize (*ZmNHX1*) and rice (*OsNHX1*), which results in enhanced sequestration of Na^+^ in the root vacuoles to prevent Na^+^ delivery to the shoot (Ashraf et al. 2018; Elkelish et al. 2019; Habibi 2017; Hasanuzzaman et al. 2011; Hawrylak-Nowak 2009; Kamran et al. 2019; Jiang et al. 2017; Subramanyam et al. 2019). Under certain circumstances, the positive action of Se in salt stress physiology could be intensified by giving this element along with silicon (Si) (Sattar et al. 2017).

In plants suffering from drought stress or heat stress, Se fertilization may sustain crop yields by alleviating ROS-induced damages via activation of cellular antioxidant systems (D’Amato et al. 2018a; Iqbal et al. 2015; Jóźwiak and Politycka 2019; Malerba and Cerana 2018; Sajedi et al. 2011). Increased activity of antioxidant enzymes and reduced lipid peroxidation have been reported in wheat, maize and cucumber plants grown under water deficit (Nawaz et al. 2016; Yao et al. 2009) and in wheat plants subjected to heat stress (Iqbal et al. 2015; Jóźwiak and Politycka 2019). Foliar Se application increased the relative water content, pigment and free amino acid accumulation, and the agronomic traits (e.g. fodder yield, crude protein, fiber, nitrogen free extract) of water-stressed maize plants (Nawaz et al. 2016), and promoted gas exchanges, Fe uptake and accumulation of osmoprotectants (total soluble sugars and free amino acids) in wheat, thus ultimately increasing grain yield (Nawaz et al. 2015,2016). Recently, high accumulation of osmolytes like proline and K^+^, and intense N metabolism have been described in maize plants cultivated in soil fertilized with Se and poorly water-irrigated (Bocchini et al. 2018). Epigenetic changes in DNA methylation in these plants caused by simultaneous water deficit and Se fertilization indicated that Se could up-regulate the expression of a number of genes implied in the plant tolerance to environmental stresses, like those coding for phytoene synthase (PSY), important enzyme for the preservation of cell carotenoids, sorbitol dehydrogenase (SDH), which controls osmolyte biosynthesis under drought stress, and alcohol dehydrogenase (ADH), functioning in plant adaptation to abiotic stress.

Selenium biofortification is also efficient in ameliorating the nutritional quality of vegetable products derived from plants grown under drought stress. For instance, olives produced by olive trees subjected to water shortage hold higher fresh weight and increased the level of phenols and pigments at harvest (D’Amato et al. 2018a). Furthermore, the extra virgin olive oil (EVOO) obtained from these olives was of superior nutritional value due to the longer shelf life and improved oxidative stability against oxidation processes.

In addition to the positive effects elicited by Se in plants subjected to abiotic stress, Se supplementation has been reported to protect plants from biotic stress caused by pathogenic fungi (Kornas et al. 2019; Xu et al. 2020) and a variety of generalist invertebrates and herbivores (Freeman et al. 2007,2009; Quinn et al. 2010; Valdez Barillas et al. 2011), suggesting that crops enriched with Se may require less synthetic fungicides and pesticides during their cultivation. Therefore, besides being applied to crops to address malnutrition in vulnerable populations, Se might be used to attain agro-ecological plant protection and sustainable use of agrochemicals. Alternatively, Se could be employed as an ecological insecticide (Mechora 2019) or fungicide (Wu et al. 2014; Xu et al. 2020). In this respect, Wu et al. (2014) proposed the use of Se in soil for the control of the postharvest disease of fruits and vegetables caused by *Penicillium expansum*, while Liu et al. (2019) and Xu et al. (2020) endorsed the use of Se for the control of the oilseed rape disease caused by *Sclerotinia sclerotiorum*.

## Impact of Se biofortification on food crop nutritional traits

### Effects of Se-biofortification on accumulation of Se species

**Fig. 4:**
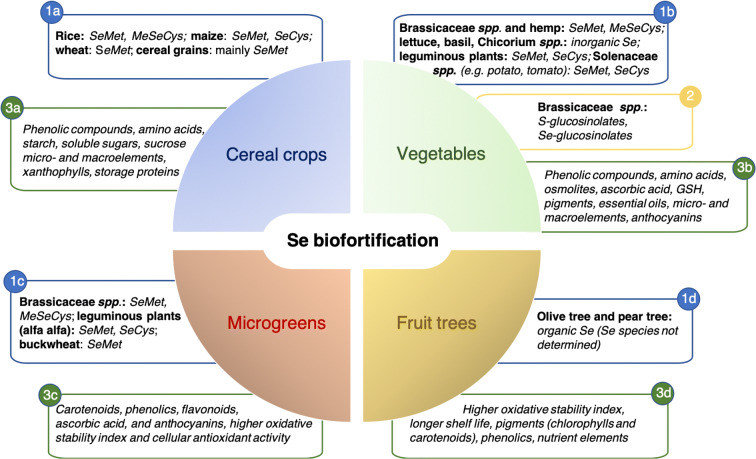
Metabolic targets of Se biofortification in terms of Se species (1a-d), glucosinolates (2, only for *Brassicaceae* spp.), other nutrients and beneficial phytochemicals (3a-d). Plants have been divided in 4 categories according to D’Amato et al. (2020): cereal crops, vegetables, microgreens and fruit trees

1Beyond achieving increases of total Se concentration in crops, one main task of Se biofortification is to enrich plants with Se compounds that provide health benefits to humans and animals (Fig. 4) (Newman et al. 2019). SeCys and SeMet are important precursors for the synthesis of mammalian selenoproteins, function as direct antioxidants in cells due to their easy oxidation and have a major role in protein repair (Rahmanto and Davies 2012). Furthermore, SeMet and other organic Se compounds like methylseleninic acid (MSA), methylselenol (MSe), γ-glutamyl-selenium-methylselenocysteine (γ-GluMeSeCys) and MeSeCys, have recognized efficacy in chemoprevention (Spallholz 2019; Tarrado-Castellarnau et al. 2015; Vinceti et al. 2018).

Crop species and varieties differ in the ability to convert inorganic Se into organic forms. Cereal crops in particular, can store Se in seeds mainly in the form of SeMet (Broadley et al. 2010), whose translocation in rice is apparently promoted by the NRT1.1B transporter (Zhang et al. 2019). Brassicaceae *spp*., including broccoli (*Brassica oleracea* L.), radish, Chinese cabbage (*Brassica rapa* L.), salad rocket and wild rocket can accumulate high amounts of organic Se species, especially SeMet and/or MeSeCys (Bachiega et al. 2016; Bañuelos et al. 2015; Ramos et al. 2011; Schiavon et al. 2016; Šindelaŕǒva et al. 2015). The same Se compounds have been identified in hemp (*Cannabis sativa* L.), rice (*Oryza sativa* L.), and potato (*Solanum tuberosum* L.) (Huang et al. 2018; Stonehouse et al. 2020; Zhang et al. 2019). Leguminous plants *spp.* like chickpea (*Cicer arietinum* L.), soybean (*Glicine max* L.), and alfalfa (*Medicago sativa* L.) accumulate SeMet and SeCys, but not methylated Se amino acids (Chan et al. 2010; Hajiboland and Amjad 2008; Poblaciones et al. 2014). Organic Se has been found in olive trees (*Olea europaea* L.), and pear trees (*Pyrus communis* L.), but the Se species have not been determined (D’Amato et al. 2018a; Deng et al. 2019). Leafy vegetables like lettuce (*Lactuca sativa* L.), basil (*Ocimum basilicum* L.) and Cichorium *spp*., instead, contain only inorganic Se (Hawrylak-Nowak 2008; Stibilj et al. 2011).

### Effects of Se-biofortification on Se-S crosstalk and accumulation of S-metabolites

One common observation from biofortification studies is that the content of inorganic or organic Se species tendentially builds up in crops fertilized with Se, while the amount of S-containing phytochemicals with functional roles in plant defense mechanisms and human health, such as glucosinolates (GLS), glutathione and S amino acids, might undergo variation depending on Se dose and species to be used, plant species and genotype, and mode of Se fertilization (foliar fertilization or soil amendment with Se) (Bachiega et al. 2016; Robbins et al. 2005; Schiavon et al. 2013; Schiavon and Pilon-Smits 2017b). GLS and their hydrolysis products (isothiocyanates, ITC) in particular, are recognized key molecules in cancer prevention, and have received great attention in biofortification studies of Brassicaceae *spp*. (Melrose 2019; Prieto et al. 2019). Increased accumulation of ITC has been recently observed in kale (*Brassica oleracea* L.) roots in response to selenite and NaCl joint application, thus making this food crop superior for chemopreventive effects (Kim et al. 2018).

The impairment of S metabolites has been frequently observed in plants supplemented with high selenate or selenite doses. This is likely because Se and S compete for the uptake and metabolic processes in plants (McKenzie et al. 2019; Schiavon and Pilon-Smits 2017a; Tian et al. 2016,2018). Negative effects of Se/S competition on the synthesis of GSH and GLS precursor amino acids, and on the expression of genes within the GLS pathway have been largely described (Dall’Acqua et al. 2019; McKenzie et al. 2019; Schiavon et al. 2016; Tian et al. 2016). Methionine (Met) is the precursor amino acid for the synthesis of aliphatic GLS, and the decrease of its content in response to Se fertilization has been associated with lower accumulation of Met-derived-GLS in salad rocket (*Eruca sativa* Mill.), radish (*Raphanus sativus* L.) and other Brassicaceae *spp*. (Dall’Acqua et al. 2019; Schiavon et al. 2016). In broccoli, discrepant results have been reported. Barickman et al. (2013) and Robbins et al. (2005) noted the decline of aliphatic GLS and sulforaphane, a GLS hydrolysis sulphur-containing aglycon byproduct, in broccoli supplied with high Se. In contrast, Sepúlveda et al. (2013) did not find any significant change in GLS and sulforaphane content, neither in myrosinase activity, in broccoli treated with high Se (100 µM selenate). Similarly, Tian et al. (2016) did not measure any appreciable variation of GLS content in broccoli sprouts exposed to 100 µM selenite or selenate, and hypothesized an equilibrium between GLS biosynthesis and hydrolysis.

Differential effects of Se application on the synthesis of S-metabolites can be observed between distinct plant species of the same genus, as recently reported in two rocket species, namely salad rocket and wild rocket (*Diplotaxis tenuifolia*) (Dall’Acqua et al. 2019). Rocket salad plants treated with 20 and 40 µM selenate exhibited reduced capacity to accumulate S amino acids, GSH and GLS, mainly because of Se/S competition processes and repression of genes involved in S reduction and GLS synthesis and hydrolysis. In contrast, wild rocket plants supplied with equal selenate concentrations accumulated more Se, without significant effects on S uptake and metabolism. Interestingly, wild rocket contained large levels of osmolytes (proline) in response to increasing Se application, likely to prevent the oxidative stress that is often generated by high Se in tissues.

Beside S-glucosinolates, Brassicaceae *spp*. supplemented with Se can produce (methylseleno)glucosinolates and their Se-containing aglycons (Matich et al. 2012,2015; McKenzie et al. 2019), whose bioactivity as anticancer and antimicrobial agents is considered higher compared to GLS (Emmert et al. 2010). The majority of Se-GSL identified so far are aliphatic, with Se in the position of the S donated by Met and not in the sulphate group or on the bond with glucose (Matich et al. 2012,2015). The Se-glucosinolates 3-(methylseleno)propyl glucosinolate, 4-(methylseleno)butyl glucosinolate, 5-(methylseleno)pentyl glucosinolate, 4-(methylseleninyl)butyl glucosinolate and seleno-2-phenylethyl glucosinolate have been described in broccoli, cauliflower and forage rape (Matich et al. 2012,2015), while 4-(methylseleno)but-3-enyl glucosinolate and related isothiocyanates (isomers of 4-(methylseleno)but-3-enyl isothiocyanate) have been tentatively identified in radish plants (McKenzie et al. 2019). In Se-enriched radish and broccoli, the up-regulation of the gene coding for APS kinase (APSK), the key branch point enzyme of GSL biosynthesis, was observed and explained as a protective mechanism activated by plants to control S uptake and limit Se-GLS production (McKenzie et al. 2017,2019). The repression of genes encoding enzymes promoting aliphatic GLS biosynthesis and the concomitant up-regulation of genes coding for enzymes involved in indole GLS generation were also hypothesized as part of the same protective mechanism that aims to generate indole GLSs over aliphatic GLS to restrict the synthesis of Se-GLS (McKenzie et al. 2019).

### Effects of Se-biofortification on accumulation of other health beneficial phytochemicals

At low doses Se is beneficial to plants and typically improves the antioxidative capacity, increases the content of nutrient elements and improves the profile of worthwhile phytochemicals. Thus, high Se food represents a valuable tool to improve the diet, nutritional quality and health in world population. Very recently, D’Amato et al. (2020) surveyed the impact of Se biofortification on the nutraceutical profile of food crops. The authors grouped them by crop category (arable crops, vegetables, microscale vegetables, and fruit trees) and highlighted the foremost effects of Se biofortification on the content of total Se, inorganic/organic Se species, and bioactive compounds, which are summarized and implemented in Fig. 4.

With respect to arable crops, the enrichment with Se mostly leads to higher antioxidant activity of their grains and greater content of nutrients, amino acids, phenols, anthocyanins, sugars, and organo-Se compounds (D’Amato et al. 2017,2019,2020; Skrypnik et al. 2019). Also, in upland rice polished grains, Se application induced higher concentration of storage proteins like albumin, globulin, prolamin, and glutelin (Reis et al. 2020).

In horticultural vegetables, Se fertilization is ascertained to augment the antioxidant activity, nitrogen and S assimilation, accumulation of pigments (carotenoids and anthocyanins), soluble phenols, and ascorbic acid (Dall’Acqua et al. 2019; Hawrylak-Nowak et al. 2008,2018; Mimmo et al. 2017; Schiavon et al. 2013,2016; Ríos et al. 2010). Enhanced production of essential oils, hydroxycinnamic acids, total phenolics, anthocyanin and antioxidant activity has been recently reported by Skrypnik et al. (2019) in sweet basil (*Ocimum basilicum* L.) leaves, while increased accumulation of flavonoid compounds, especially naringenin chalcone and kaempferol, and several amino acids except proline, was observed in Se-biofortified tomato fruits and radish roots, respectively (Schiavon et al. 2013,2016).

Seedlings of edible vegetables supplemented with selenate seem to preferentially accumulate organic Se, and their nutritional value is generally ameliorated by Se enrichment (D’Amato et al. [Bibr CR39][Bibr CR41]; Islam et al. 2020; Pannico et al. 2020). In fact, Ávila et al. (2014) screened Se-enriched cultivars from the six largely consumed Brassica vegetables and found that all were able to accumulate MeSeCys and contained higher levels of GLS, especially glucoraphanin. Increased accumulation of several bioactive compounds (carotenoids, phenolics, flavonoids, ascorbic acid, and anthocyanins) was observed in Se-biofortified wheat microgreens (Islam et al. 2020). Similarly, Se fertilization induced larger accumulation of total phenolics in coriander (*Coriandrum sativum* L.), green basil, purple basil, and tatsoi (*Brassica narinosa* L.) microgreens grown in soilless cultivation (Pannico et al. 2020), while Se-enriched chickpea sprouts were proved to be a valuable source of oil characterized by high oxidative stability index (OSI) and cellular antioxidant activity (CAA), due to reduced lipase and lipoxygenase (LOX) activities and increased content in phenolics, respectively (Guardado-Félix et al. 2019). Interestingly, Puccinelli et al. (2019) suggested the production of Se-enriched sprouts from seeds collected from plants fertilized with Se as a novel biofortification approach.

Little information is available about the effects of Se on fruit trees (D’Amato et al. 2020). In the few studies accomplished in this area of research, spraying leaves or fruits with Se was the only approach used to enrich fruits (e.g. pears, peach, apples, olives) with Se (D’Amato et al. [Bibr CR37][Bibr CR38][Bibr CR39]; Deng et al. 2019; Pezzarossa et al. 2012). Se foliar fertilization of olive trees increased the accumulation of several nutritional components of olives and EVOO, such as mineral elements, pigments (carotenoids and chlorophylls) and phenolic compounds (e.g. oleacein, ligustroside aglycone, and oleocanthal) (D’Amato et al. [Bibr CR37][Bibr CR38]). Such results indicate that Se fertilization might be used to increase the nutritional value of EVOOs in order to meet the quality standards required by the European Food Safety Authority (EFSA) (D’Amato et al. 2020).

## Impact of high Se food on emerging viral epidemics

Selenium deficiency in humans is associated with a variety of pathological conditions, including a cardiomyopathy endemic in China termed Keshan disease, reduced male fertility, mood disorders, and disturbance of thyroid functions (Rayman 2012,2020). The Se nutritional status of individuals also impacts on the immune system defenses activated to combat viral infections and on the viral pathogen itself (Guillin et al. 2019; Harthill 2011). If Se level in blood is below 1µM, the metabolic oxidative stress generated in the host cells due to decreased activity of selenoproteins, especially glutathione peroxidases (GPX), may lower the immune system and trigger genome mutations in the parasitic virus that generally change the virus from being benign or mildly pathogenic to become highly virulent (Beck et al. 2000; Nelson et al. 2001). Such RNA viral mutations are faster and long-lived in Se-deficient than in healthy individuals (Harthill 2011).

Se-deficient infected hosts get benefits from Se supplementation, which result in decrease of virus mutation frequency and load, and enhanced immune competence with better outcomes for viral infections (Beck et al. 2003; Harthill 2011). This has been corroborated for at least influenza virus type A and Coxsackievirus B3 (CVB3), human immunodeficiency virus/acquired immunodeficiency syndrome (HIV/AIDS) (Beck et al. 1995; Broome et al. 2004; Kupka et al. 2004; Nelson et al. 2001). Nonetheless, mutated virulent RNA viruses persist as pathogenic and infectious parasites in the hosts and can be transferred to individuals with Se-sufficient status (Harthill 2011).

Very recently, an epidemic caused by a novel coronavirus (COVID-19 or 2019‐CoV) belonging to the same group of β-coronaviruses as severe acute respiratory syndrome (SARS) in 2002 and Middle East respiratory syndrome (MERS) (Cohen and Normile 2020), is spreading worldwide while threating human health and threatening the world economy. So far, no pharmacological treatment or vaccine exist. In any case, pharmaceutical drugs are usually ineffective against RNA viruses (Lyons 2019), while vaccines against coronaviruses carry an unacceptable risk of paradoxical immune enhancement (Tirado and Yoon 2003; Bolles et al. 2011; Ricke and Malone 2020) Therefore, alternative or supplementary natural treatments should be considered to reduce the viral load in the hosts and enhance their immune system. Similar to Zn, Se supplementation to COVID‐19 infected people with low Se blood levels could be an option to explore as a natural treatment of this virus (Zhang and Liu 2020). In this context, Se biofortification programs attain incremental importance, as Se enriched food crop is higher in nutritional components compared to Se intake via tablets, and thus provides more benefits to infected consumers. If the food is additionally enriched with Zn and Fe besides Se, its potential in contrasting viral infection could be significantly increased.

## Conclusions and future prospects

The element Se may serve in different agrotechnological applications. Among them, agronomic biofortification through Se fertilizers and conventional breeding are likely the most widespread and accepted methods to combat the Se deficiency worldwide. Circular systems where Se phytoremediation is combined with biofortification strategies and genetic engineering of crops, although promising in the context of sustainable agriculture, are still poorly developed because of some practical limitations. In contrast, nanosized Se biofortification is increasingly gaining attention from scientists. Further investigation is needed to understand if the use of SeNPs is safe to consumers, and which chemical modifications they might undergo in the environment and during food processing. Studies on crop biofortification with multiple micronutrients and the use of PGPR in agronomic biofortification are only at the beginning, but might represent a future and promising area of Se biofortification research.

Selenium biofortification primarily aims to enrich crops with Se-species and antioxidant compounds with positive impact on human (and animal) nutrition and health. The role of Se-enriched food in preventing or contrasting viral infections in particular, is of great relevance in the recent scenario of a viral disease pandemic and represents a field of research that deserves a broad investigation. Se biofortification could also be used to increase crop yield under sub-optimal conditions, mitigating the negative effects of such environments on plant physiology, while increasing plant antioxidant properties and content in healthy phytochemicals. On this account, studies attempting optimization of Se biofortification strategies for improving food crop nutritional under challenging environments are of great interest on a global scale.
